# The aniline-to-azobenzene oxidation reaction on monolayer graphene or graphene oxide surfaces fabricated by benzoic acid

**DOI:** 10.1186/1556-276X-8-372

**Published:** 2013-09-02

**Authors:** Myungjin Lee, Kijeong Kim, Hangil Lee

**Affiliations:** 1 Department of Chemistry, Sookmyung Women’s University, Seoul 140-742, Republic of Korea; 2 Beamline Research Division, Pohang Accelerator Laboratory (PAL), Pohang 790-784, Republic of Korea

**Keywords:** Oxidation reaction, GO surface, Raman spectroscopy, HRPES, Reaction reagent

## Abstract

The oxidation of aniline to azobenzene was conducted in the presence of either monolayer graphene (EG) or graphene-oxide-like surface, such as GOx, under ultra-high vacuum conditions maintaining a 365-nm UV light exposure to enhance the oxidation reaction. The surface-bound products were investigated using micro Raman spectroscopy, high-resolution photoemission spectroscopy, and work function measurements. The oxygen carriers present on the GOx surfaces, but not on the EG surfaces, acted as reaction reagents to facilitate the oxidation reaction from aniline to azobenzene. Increasing the aniline concentration at 300 K confirmed that the exchange ratio from the aniline to the azobenzene was enhanced, as determined by the intensity ratio between the aniline- and azobenzene-induced N 1 *s* core-level spectra. The work function changed dramatically as the aniline concentration increased, indicating that the aniline on the GOx surface conveyed *n*-type doping characteristics at a low coverage level. A higher aniline concentration increased the *p*-type doping character by increasing the azobenzene concentration on the GOx surface. A comparison of the oxidation reactivity of aniline molecules on the EG or GOx surfaces revealed the role of the oxygen carriers on the GOx surfaces in the context of catalytic oxidation.

## Background

Among the various systems composed only of carbon 5atoms, graphene (a two-dimensional allotrope of carbon) presents a framework for understanding of the properties of all other carbon allotropes. The properties of graphene, including a high intrinsic mobility [1,2], a large theoretical specific surface area, and a high chemical stability, are potentially useful in applications ranging from chemical sensors to transistors [3-8]. Toward exploiting these unique properties of graphene, several research groups have attempted to fabricate large-scaled graphene oxide sheets [9-12]. Graphene oxide (GO) is a layered material consisting of hydrophilic oxygenated graphene oxide sheets bearing oxygen functional groups on their basal planes and edges [13]. It is a useful platform for fabricating functionalized graphene that can potentially confer improved mechanical, thermal, or electronic properties. The numerous chemical functionalities on a GO surface are expected to readily lend themselves to further chemical functionalization. Graphene-based materials, therefore, show promise in a variety of technological applications. The use of GO surfaces as catalysts of synthetic transformations is a relatively new research area with outstanding potential. Current efforts are directed toward harnessing the oxygen carriers present on GO surfaces as heterogeneous catalysts [14-16].

In this study, we systematically compared and investigated the oxidation of aniline to form azobenzene on monolayer graphene (EG) or graphene-oxide-like (GOx) surfaces fabricated with benzoic acid. Moreover, we focus on examining the difference between EG and GOx surfaces in one substrate, simultaneously. Raman spectroscopy and high-resolution photoemission spectroscopy (HRPES) were used to characterize the surface-bound products. The carboxyl groups introduced onto the graphene surface upon oxidation by benzoic acid to GOx allowed aniline to react with the oxygen carriers. The oxidation of aniline proceed *via* a reaction between the aniline amine groups and the oxygen groups on the GOx surface under ultra-high vacuum (UHV) conditions maintaining a 365-nm UV light exposure.

Generally, it is hard to distinguish the difference between EG and GOx surfaces in one substrate due to the large size of the HRPES beam. Hence, no previous systematic experimental studies have examined the oxidation of aniline on a GOx surface. However, this study is meaningful with regards to indicating this distinctive difference using the feature of micro Raman spectroscopy.

## Methods

A Si-terminated 6H-SiC(0001) substrate (Cree Research, Durham, NC, USA) was used to fabricate EG. The substrate was degassed, annealed at 1,200 K under a Si flux (1 Å/min), and graphitized at temperatures up to 1,500 K (for 2 min) to produce a monolayer of graphene (EG). The annealing temperature was monitored using an infrared pyrometer (with an emissivity of 0.9).

**Figure 1:**
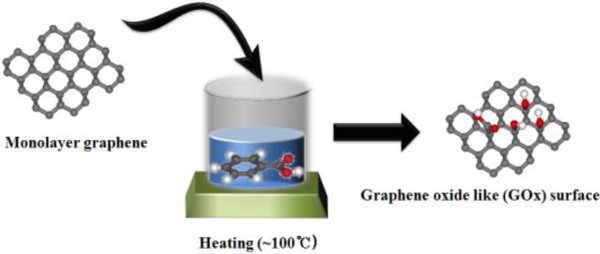
**The method how fabricating graphene-oxide-like (GOx) surface.** The scheme indicates that the fabrication of the GOx surfaces using benzoic acid.

A GOx surface was fabricated by exposing the EG surface to benzoic acid (Sigma Aldrich, purity, 97%, St. Louis, MO, USA). As shown in Figure 1, the EG surface structure was confirmed using LEED and C 1 *s* core-level spectroscopy (not shown here) and then immersed into a benzoic acid solution for 3 min. The surface was subsequently reintroduced into the UHV chamber.

Aniline (Sigma Aldrich, purity, 99.9%) was purified by turbo pumping to remove impurities prior to dosing onto the GOx surfaces. A direct doser, controlled by means of a variable leak valve, was used to dose the substrates. Raman spectra of the samples were collected using a home-built system equipped with an Ar^+^ ion laser (Spectra-Physics Stabilite 2017, Santa Clara, CA, USA) as an excitation source; a spectrometer (Horiba Jobin Yvon TRIAX 550, Kyoto, Japan), and a CCD detector (Horiba Jobin Yvon Symphony) cooled to 140 K. The wavelength of the incident excitation beam was 514.5 nm. HRPES experiments were performed at the 8A2 beamline at the Pohang Accelerator Laboratory, which was equipped with an electron analyzer (SES100, Gamma Data Scienta, Uppsala, Sweden). The N 1 *s* core-level spectrum was obtained using photon energies of 460 eV. Secondary electron emission spectra (−20 V sample bias) and valence band spectra were measured at photon energies of 80 eV. The binding energies of the core-level spectra were determined with respect to the binding energies of the clean Au 4*f* core level and the valence band (Fermi energy) for the same photon energy. All spectra were recorded in the normal emission mode. The photoemission spectra were carefully analyzed using a standard nonlinear least-squares fitting procedure with Voigt functions [17].

## Results and discussion

**Figure 2:**
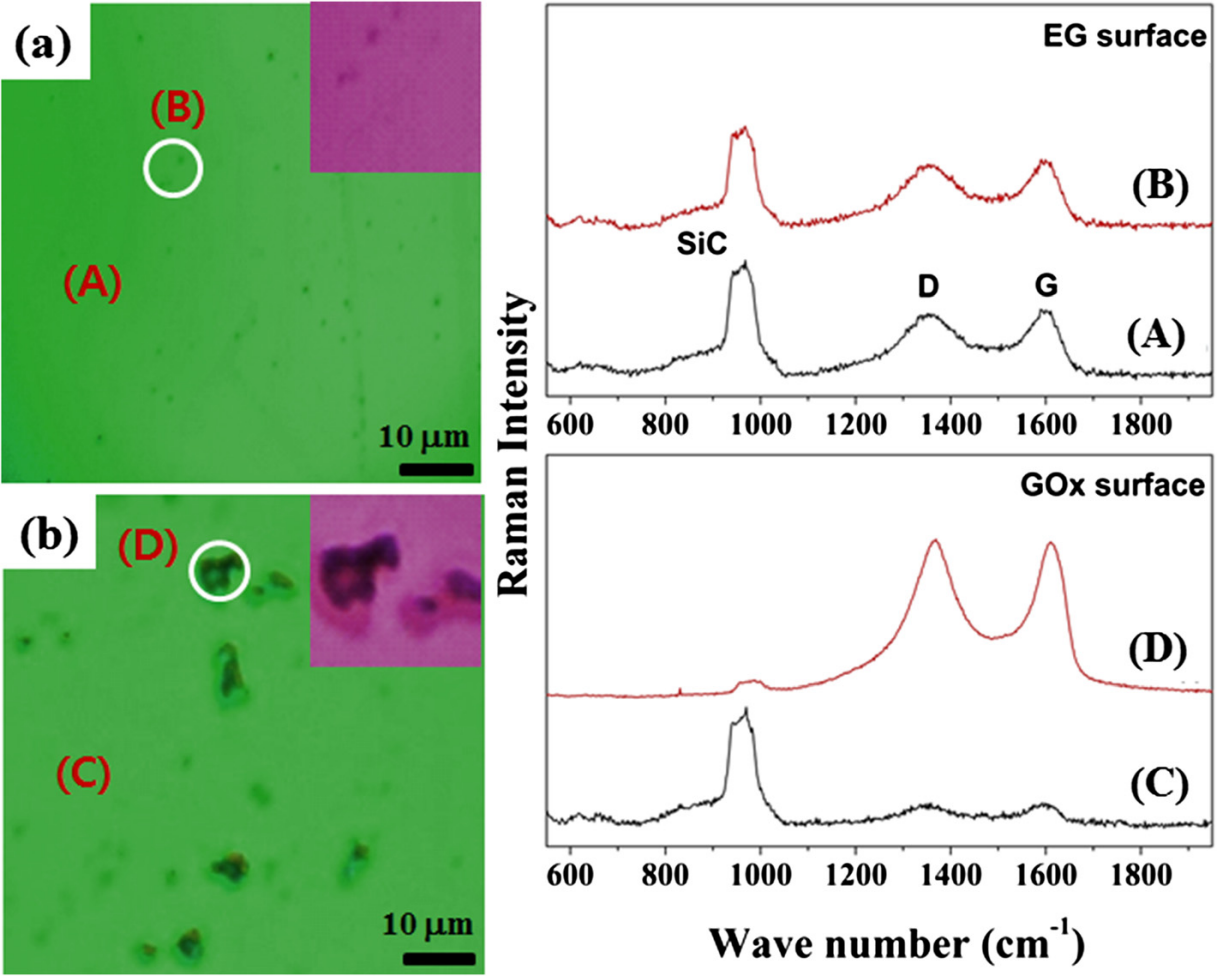
**The micro optical images obtained by the Raman spectra.** Micro optical images of **(a)** monolayer EG and **(b)** a GOx surface. Top graph illustrates the Raman spectra obtained from the bottom position (curve A) or the small-particle position on the EG (curve B). (d) Bottom graph illustrates the Raman spectra acquired from the bottom (curve C) and the particle position (curve D) of the GOx surface. The inset images show magnified views of the areas indicated by the white circles.

Raman spectroscopy, which is sensitive to the chemical functional groups on a surface, is a useful tool for comparing the properties of the EG and GOx surfaces. Optical microscopy images of the EG (a) and GOx (b) surfaces were acquired, and their corresponding Raman spectra at two positions (over a particle and over the bottom region) were collected, as shown in Figure 2. Figure 2a shows the optical microscopy image of the EG surface grown on a 6H-SiC(0001) substrate. The EG surface appeared clean, with a few small particles remaining (not oxide). The conditions of the surfaces were assessed by collecting the Raman spectra in a bottom region (marked (A)) and at a particle (marked (B)). A comparison of the D and G Raman bands revealed similar spectra that were characteristic of the EG surface. Note that the G band values (1,597.6 cm^–1^ and 1,597.9 cm^–1^) were indistinguishable from the G band position of graphene. The ratio of the D and G band intensities, ID/IG, corresponded to the average value for graphene. The Raman D/G intensity ratios at both the bottom and small particle positions on the EG surface were 0.73, indicating that the surface properties at either position were typical of an EG surface [16].

Figure 2b shows an optical image of a GOx surface that had been freshly fabricated by treatment with benzoic acid (see Figure 1). Contrasting with Figure 2a, the GOx surface clearly displayed two regions: a bottom region and a particle region. As with the EG surface, the Raman spectra were collected at these two positions. As expected, the particle position (marked (D)) yielded a distinct Raman spectrum, whereas the bottom position (marked (C)) displayed a typical EG surface spectrum, with the G band at 1,597.6 cm^–1^. Figure 2f shows that the graphene oxide spectrum was measured with a high intensity. Note that the G band (1,613.1 cm^–1^) obtained from the particle position was shifted toward higher wavenumbers relative to the G bands of graphene and graphite. The ratio of the D and G band intensities, ID/IG, is inversely proportional to the average size of the *sp*^2^ domains. The Raman D/G intensity ratio for the GOx surface was found to be 0.92, similar to the results reported previously for graphene oxide [18].

A Raman spectrum similar to the spectrum of GO surface indicated that benzoic acid treatment successfully yielded a GOx surface. The EG and GOx surfaces were used in the subsequent experiments involving the oxidation of aniline, which is difficult to oxidize in general. We hypothesized that only the GOx surface would be able to oxidize aniline if the oxidation process is possible. Because the oxidation of aniline on a GOx surface could not be fully characterized by micro Raman spectroscopy alone, we obtained the core-level spectra of the N 1 *s* peak, which is an indicator of the overall molecular electronic properties. The morphological discrepancies observed between the optical images could only be explained in terms of a surface reaction, as supported by the HRPES results.

**Figure 3:**
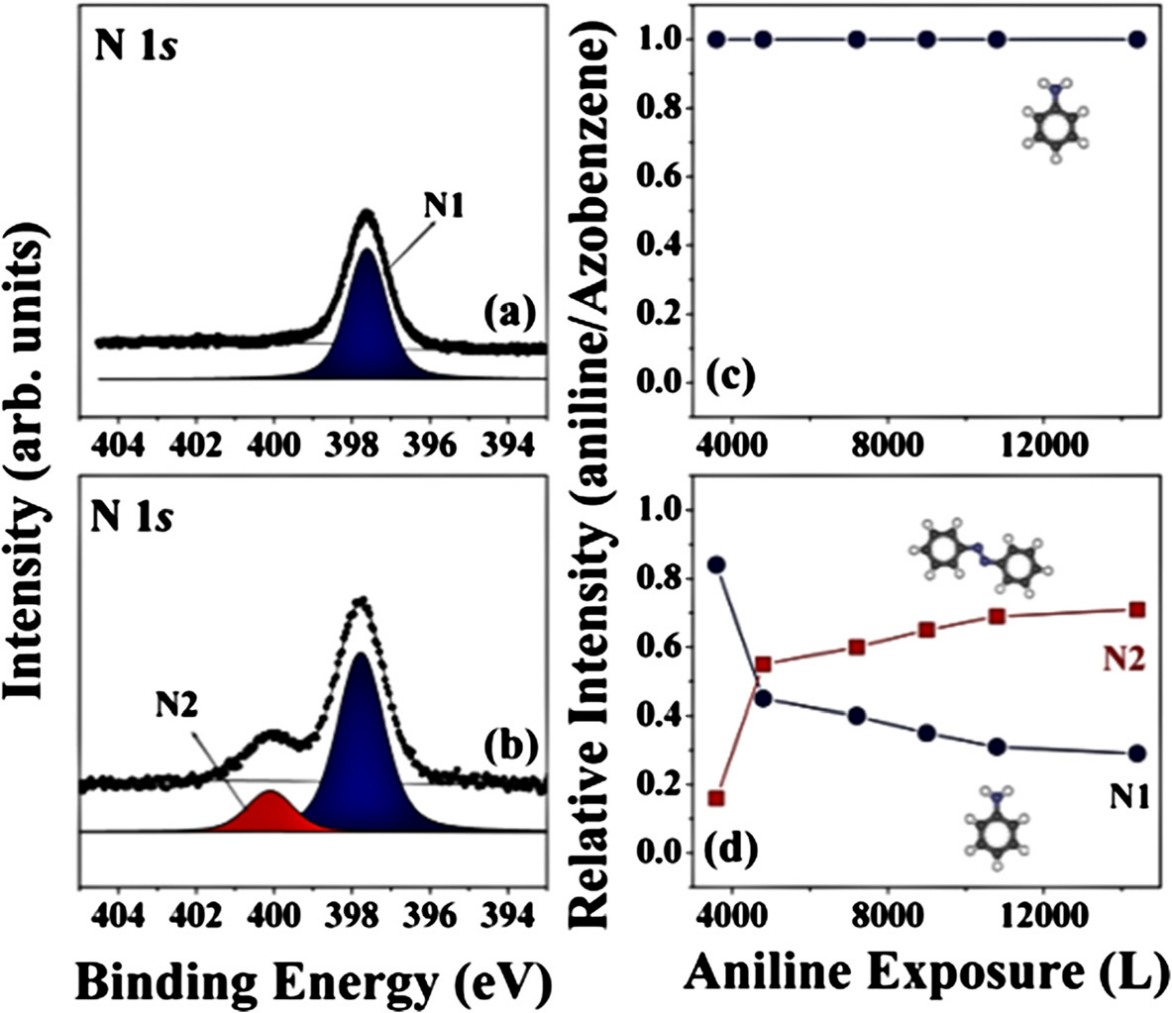
**HRPES measurements indicating oxidation from aniline to azobenzene on GOx surfaces prepared using benzoic acid.** N 1 *s* core level spectra of **(a)** 3,600 L aniline on EG at 300 K, **(b)** 3,600 L aniline on a GOx surface prepared using benzoic acid at 300 K. The N1 and N2 peaks corresponded to the aniline and azobenzene nitrogen peaks. **(c)** and **(d)** show the plots of the intensity ratio between the N1 and N2 features as a function of the aniline coverage on the EG and GOx surfaces, respectively.

Figure 3 shows the surface-sensitive N 1 *s* core-level spectra of aniline on the EG and GOx surfaces, obtained using HRPES at 460 eV photon energy. The N 1 *s* core spectra of 3,600 L aniline on EG or on GOx surfaces were obtained first. As expected, the presence of aniline resulted in low-intensity nitrogen peaks on the EG surface because the EG surface was too inert to react to the oxidation of aniline, illustrated in Figure 3a. The N 1 *s* core-level spectrum was then obtained after preparing a sample to have 3,600 L aniline on the GOx surface. Two distinct nitrogen peaks corresponding to the aniline peak (NH_2_ is marked N1) and azobenzene peak (NO_2_ is marked N2) clearly appeared, as shown in Figure 3b, indicating that the oxidation reaction had proceeded as we expected. The relative intensity ratio of the N 1 *s* aniline peak (N1) was found to be 0.84 at 397.8 eV, and the ratio of the azobenzene peak (N2) was 0.16 at 400.1 eV, for a 3,600-L aniline sample on the GOx surface [19,20]. These N 1 *s* peaks indicated that aniline had oxidized to azobenzene in the presence of the oxygen groups on the GOx surface, which suggested that the GOx surface acted as a reaction reagent at 300 K. The oxidation reaction efficiency under a 365-nm UV light exposure was measured as the aniline coverage was increased from 3,600 L to 14,400 L.

The plots of the coverage-dependent intensity of the aniline peaks (N1) and the azobenzene peaks (N2) on the EG and GOx surfaces are displayed in Figure 3c,d. Figure 3c shows that the intensity ratio remained unchanged, although the exposure of aniline was increased to 14,400 L. Thus, we concluded that the EG surface did not promote the oxidation reaction process because oxygen groups were not present. Figure 3d, on the other hand, clearly revealed that the relative intensity ratio between aniline and azobenzene increased with increasing aniline coverage on the GOx surface. As the aniline coverage increased from 3,600 L to 14,400 L aniline, the azobenzene (N2) peak increased significantly from 0.16 to 0.71 whereas the aniline (N1) peak decreased from 0.84 to 0.29. These results suggested that the high concentration of aniline enhanced the occurrence of azobenzene due to the Le Chatelier's principle on the GOx surface. It can be clearly explained that as the aniline coverage increased, the oxidation reaction involving the oxygen carriers on the GOx surface proceeded with greater efficiency because the high aniline coverage increased the possibility of the oxidation reaction. Table 1 summarizes the aniline and azobenzene intensity measurements as a function of the aniline surface coverage.

**Table 1:** Intensity measurements indicating relative aniline and azobenzene coverage

**Aniline exposure (L)**	**Relative intensity of aniline (N1)**	**Relative intensity of azobenzene (N2)**
3,600	0.84	0.16
4,800	0.45	0.55
7,200	0.40	0.60
9,000	0.35	0.65
10,800	0.31	0.69
14,400	0.29	0.71

A function of aniline surface coverage at 300 K.

**Figure 4:**
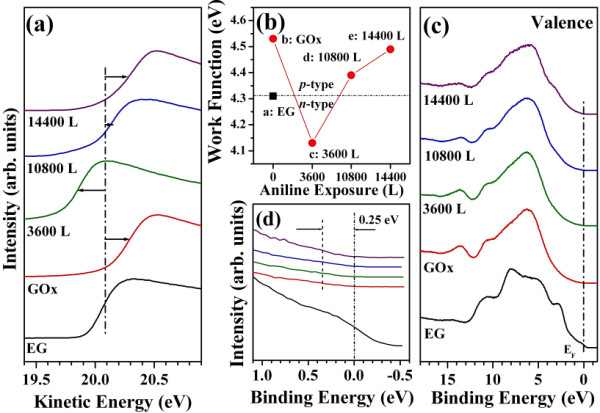
**The several data acquired from HRPES experiments. (a)** Work function measurements and **(b)** a plot of the work function values for each sample (a: monolayer EG, b: GOx surface, c: 3,600 L aniline, d: 10,800 L aniline, e: 14,400 L aniline). **(c)** Valence band spectra of the five samples. Black curve, monolayer EG; red curve, GOx surface prepared using benzoic acid; green curve, 3,600 L aniline; blue curve, 10,800 L aniline; and purple, 14,400 L aniline. **(d)** The magnified Fermi edge spectrum, which corresponds to Figure 4c.

The work function was measured as the center position of the low kinetic energy cut-off for each sample, as shown in Figure 4a. The monolayer EG spectrum (the black spectrum in Figure 4a) yielded a work function of 4.31 eV [20,21]. Because the N 1 *s* core-level spectra had confirmed that aniline did not react to form azobenzene on the EG surface, the work function measurements were conducted only on the GOx surface, for various aniline coverage levels. The red spectrum in Figure 4a shows the work function of the GOx surface, showing that the secondary electron edge had shifted by 220 meV (*Δϕ* = 0.22 eV) toward higher kinetic energies relative to the monolayer EG secondary electron edge. This result indicated that the oxygen carriers on the GOx surface acted as *p*-type dopant materials. After measuring the GOx surface work function, a 3,600 L aniline coverage was deposited at 300 K (the green spectrum in Figure 4a) on the GOx surface. Interestingly, this spectrum showed that the secondary electron edge had shifted by 300 meV (*Δϕ* = −0.30 eV) toward lower kinetic energies relative to the pristine monolayer EG, indicating *n*-type doping due to aniline. The amine group in the aniline donated an electron carrier to the GOx surface, indicating that aniline acted as an electron dopant on the EG surface (*n*-type characteristic). The blue spectrum in Figure 4a shows the secondary electron edge obtained after deposition of 10,800 L aniline at 300 K. Because the oxidation reaction proceeded more extensively at this exposure level, the edge was shifted by 80 meV (*Δϕ* = 0.08 eV) toward higher kinetic energies relative to the pristine monolayer EG. Unlike aniline, azobenzene acted as an electron acceptor (*p*-type characteristic). The presence of azobenzene on the GOx surface resulted in *p*-type doping carriers. Because aniline and azobenzene were in competition on the GOx surface, the secondary electron edge did not show a significant shift toward higher kinetic energies. Finally, the aniline coverage level was increased to 14,400 L at 300 K (the purple spectrum in Figure 4a). The secondary electron edge was shifted by 180 meV (*Δϕ* = 0.18 eV) to higher kinetic energies relative to the pristine monolayer EG. This surface yielded a work function that resembled the work function of the GOx surface. These results could be readily explained in terms of the aniline coverage. At higher coverage, the reaction rate increased, thereby facilitating the oxidation of aniline to azobenzene. Figure 4b shows the dramatic change in the work function as a function of the aniline coverage.

Figure 4c shows the valence band spectra of the five samples. The spectra are colored as in Figure 4a. The black spectrum corresponds to the valence band spectrum of the monolayer EG obtained at 80 eV. The five distinct peaks corresponding to monolayer EG were clearly observed [22]. The magnified Fermi edge spectrum (Figure 4d) revealed the typical characteristics of monolayer EG. The red spectrum, obtained from the GOx surface, displayed remarkable insulating properties, as demonstrated by the band gap at 0.25 eV. The magnified valence band spectra indicated the presence of a band gap, and the insulating properties resulted from the high oxide character of the substrate. Other spectra were obtained after depositing at various coverages. These figures showed that the valence band spectra were similar to the spectra obtained from the GOx surface, even at higher coverage deposition. The oxidation process did not appear to affect the structure of the GOx surface, suggesting that the oxygen groups present on the GOx surface supplied oxygen atoms during the oxidation reaction.

The Raman spectra and HRPES experiments further supported the conclusion that the oxidation reaction occurred on the GOx surface. The work function of the surface was monitored as the doping characteristic changed from *p*-type to *n*-type due to charge transfer from the GOx surface to the adsorbed aniline or azobenzene. The doping characteristic changed from *n*-type to *p*-type as the oxidation reaction proceeded from aniline to azobenzene.

## Conclusions

The oxidation of aniline to azobenzene was investigated on a GOx surface prepared using benzoic acid. Micro optical images and their corresponding Raman spectra, HRPES measurements, and work function measurement were conducted from the samples prepared under a variety of conditions. The Raman images revealed the structure of the GOx surface prepared using benzoic acid. The HRPES measurements indicated that the relative concentration of aniline and azobenzene varied with the aniline surface coverages. The work functions of the samples were measured as a function of the aniline surface coverage to identify the major product of the surface reaction. *n*-Type doping was observed at high aniline concentrations (at lower aniline deposition), whereas *p*-type doping was observed at high azobenzene concentrations (at higher aniline deposition) on the GOx surface. The oxygen carriers present on the GOx surface were found to act as the reaction reagents.

## Abbreviations

EG: Monolayer graphene; GOx surface: Graphene-oxide-like surface; UHV: Ultra-high vacuum; HRPES: High-resolution photoemission spectroscopy.

## Competing interests

The authors declare that they have no competing interests.

## Authors’ contributions

ML participated in overall experiments. KK conducted HRPES experiments, and HL who is a corresponding author participated in overall experiments. All authors read and approved the final manuscript.
